# KPU-300, a Novel Benzophenone–Diketopiperazine–Type Anti-Microtubule Agent with a 2-Pyridyl Structure, Is a Potent Radiosensitizer That Synchronizes the Cell Cycle in Early M Phase

**DOI:** 10.1371/journal.pone.0145995

**Published:** 2015-12-30

**Authors:** Kohei Okuyama, Atsushi Kaida, Yoshiki Hayashi, Yoshio Hayashi, Kiyoshi Harada, Masahiko Miura

**Affiliations:** 1 Section of Oral Radiation Oncology, Department of Oral Health Sciences, Graduate School of Medical and Dental Sciences, Tokyo Medical and Dental University, 1-5-45 Yushima, Bunkyo-ku, Tokyo, 113–8549, Japan; 2 Section of Maxillofacial Surgery, Department of Maxillofacial and Neck Reconstruction, Graduate School of Medical and Dental Sciences, Tokyo Medical and Dental University, 1-5-45 Yushima, Bunkyo-ku, Tokyo, 113–8549, Japan; 3 Department of Medicinal Chemistry, Tokyo University of Pharmacy and Life Sciences, 1432–1 Horinouchi, Hachioji, Tokyo, 192–0392, Japan; AntiCancer Inc., UNITED STATES

## Abstract

KPU-300 is a novel colchicine-type anti-microtubule agent derived from plinabulin (NPI-2358). We characterized the effects of KPU-300 on cell cycle kinetics and radiosensitization using HeLa cells expressing the fluorescent ubiquitination-based cell cycle indicator (Fucci). Cells treated with 30 nM KPU-300 for 24 h were efficiently synchronized in M phase and contained clearly detectable abnormal Fucci fluorescence. Two-dimensional flow-cytometric analysis revealed a fraction of cells distinct from the normal Fucci fluorescence pattern. Most of these cells were positive for an M phase marker, the phosphorylated form of histone H3. Cells growing in spheroids responded similarly to the drug, and the inner quiescent fraction also responded after recruitment to the growth fraction. When such drug-treated cells were irradiated in monolayer, a remarkable radiosensitization was observed. To determine whether this radiosensitization was truly due to the synchronization in M phase, we compared the radiosensitivity of cells synchronized by KPU-300 treatment and cells in early M phase isolated by a combined method that took advantage of shake-off and the properties of the Fucci system. Following normalization against the surviving fraction of cells treated with KPU-300 alone, the surviving fractions of cells irradiated in early M phase coincided. Taken together with potential vascular disrupting function *in vivo*, we propose a novel radiosensitizing strategy using KPU-300.

## Introduction

Microtubules, which consist of polymers of α- and β-tubulins, are major cytoskeletal components that play an important role in the regulation of chromosome separation in mitosis [1–3]. Consequently, the dynamic polymerization/depolymerization process of microtubules is a useful target in cancer therapy. Many anti-microtubule agents have been developed; in general, they can be classified into three types depending on the binding site in β-tubulin: the taxane site, the vinca domain, and the colchicine site. Drugs belonging to the former two types, such as paclitaxel and vincristine, have been already extensively used as chemotherapeutic agents in the clinic [4, 5]. Indeed, plethora of studies reported that these drugs induce mitotic arrest at early M phase, resulting in mitotic catastrophe for many types of tumor cells [6–9]. Several drugs with colchicine-like activities have been recently developed; these compounds also exert vascular disruption [10] in addition to their cytotoxic effects on tumor cells themselves. Agents of this type include combretastatin A-4 (CA-4) [11] and plinabulin (NPI-2358) [12, 13]. A phase I clinical study revealed that plinabulin is well tolerated [14, 15]. Hayashi et al. synthesized a large number of plinabulin derivatives, including KPU-300, which has a simpler 2-pyridyl structure in place of the *tert*-butylimidazole moiety that lacks the imidazole moiety, a higher affinity to β-tubulin, and higher toxicity than the parent compound [16]. The simpler structure of KPU-300 makes chemical synthesis much easier.

Radiotherapy (RT) is a powerful cancer treatment modality; however, radiosensitization of radioresistant tumor cells is necessary for efficient tumor control. Many studies have reported radiosensitization using taxane-type anti-microtubule agents [17–24]. Because such drugs synchronize many types of tumor cells in M phase, which is the most radiosensitive stage of the cell cycle [25–27], appropriate timing of administration results in optimal radiosensitization. On the other hand, because some cells are radiosensitized without undergoing mitotic arrest, inhibition of DNA repair is also thought to be involved in the mechanism of radiosensitization by such drugs [28–30]. However, vascular disruption has been primarily reported in the context of colchicine-type agents [31–33]. Hence, we sought to test KPU-300 as a radiosensitizer *in vitro* and to characterize its radiosensitizing mechanism. Currently, it remains unclear whether the radiosensitivity of cells accumulated in early M phase by anti-microtubule agents is consistent with that of cells in early M phase. Indeed, until recently, this question was technically impossible to address. In this study, we used the fluorescent ubiquitination-based cell cycle indicator (Fucci) system, in which cells emit red fluorescence in G1 phase and green fluorescence in S/G2/M phases [34]. By combining the Fucci system with the shake-off method, which concentrates mitotic cells [27], we could specifically collect cells in early M phase and compare their radiosensitivity with cells synchronized by KPU-300 treatment. We show here that the radiosensitivity coincides and propose a novel radiosensitizing strategy using KPU-300.

## Materials and Methods

### Cell lines and culture conditions

HeLa cells expressing the Fucci probes (HeLa-Fucci cells) were provided by RIKEN BioResource Center through the National Bio-Resource Project of MEXT, Japan. Cells were maintained in DMEM (Sigma-Aldrich, St. Louis, MO) containing 1000 mg/L glucose, supplemented with 10% fetal bovine serum (FBS) and 100 units/ml penicillin and 100 μg/ml streptomycin, at 37°C in a humidified atmosphere of 95% air and 5% CO_2_. For cell viability assays, HeLa (with no Fucci probes), SAS (human tongue cancer), HSC3 (human tongue cancer), DLD-1 (human colon cancer), Li-7 (human hepatocellular carcinoma), ACNH (human renal cell carcinoma), TE8 (human esophageal cancer), and Lu65 (human lung giant cell carcinoma) cells were obtained from the Cell Resource Center for Biomedical Research (Sendai, Japan). HeLa and TE8 cells were maintained in DMEM containing 1000 mg/L glucose, and SAS and HSC3 cells were maintained in DMEM containing 4500 mg/L glucose. ACNH, DLD-1, Li-7, and Lu65 cells were maintained in RPMI-1640 (Gibco, Grand Island, NY). All media were supplemented with 10% FBS, 100 units/ml penicillin, and 100 μg/ml streptomycin, and cultured under the same conditions as for HeLa-Fucci cells.

### Drug preparation and treatment

KPU-300, a yellow powdery substance, was developed as described previously [16]. It was stored in aliquots at -80°C at a stock concentration of 10 mM in dimethyl sulfoxide. The solution was diluted to the indicated final concentrations in the growth media described above and protected from light. Cells were irradiated using an RX-650 Cabinet X-radiator system (Faxitron, Lincolnshire, IL) at a dose rate of 0.8 Gy/min (130 kVp, 5 mA, 0.5 mm Al filtration) before or after KPU-300 treatment.

### Immunofluorescence staining

Cells grown on Lab-Tek Chamber slides (Nunc, Rochester, NY) were treated with 30 nM KPU-300 for 16 h. After treatment, cells were fixed in 4% paraformaldehyde for 30 min. Fixed cells were then incubated in rabbit monoclonal anti–β-tubulin antibody (1:50) (Cell Signaling Technologies, Beverly, MA) for 1 h at room temperature. After extensive washing in Tris-buffered saline plus Triton X-100 (TBS-T), cells were incubated with Alexa Fluor 647–conjugated anti-rabbit IgG (1:500) (Life Technologies, Carlsbad, CA) for 30 min. Finally, chamber slides were washed in TBS-T and mounted with ProLong Gold Antifade Reagent (Life Technologies) containing DAPI. Fluorescence and phase contrast images were observed using an FV10i-DOC confocal laser scanning microscope (Olympus, Tokyo, Japan) with a UPLSAPO 60× W objective lens.

### Cell viability assay

HeLa, SAS, HSC3, DLD-1, Li-7, ACNH, TE8, and Lu65 cells were plated in 96-well plates. KPU-300 was added at the indicated concentrations in quintuplicate wells. Cell viability after 24 h KPU-300 treatment was determined based on absorbance at a wavelength of 450 nm using the Cell Counting Kit 8 (DOJINDO, Kumamoto, Japan) (CCK-8).

### Time-lapse imaging

Time-lapse images were acquired using a BIOREVO BZ-9000 fluorescence microscope (KEYENCE, Osaka, Japan) at 2 h intervals for 24 h. During imaging, cells were held in a small incubation chamber (Tokai Hit, Fujinomiya, Japan) at 37°C in a humidified atmosphere containing 95% air and 5% CO_2_.

### Colony-forming assay

Cells were treated with the indicated concentrations of KPU-300 for the indicated times. For combined treatment, drug treatment was followed by X-irradiation, or irradiation was followed by drug treatment for 24 h. Immediately after combined treatment, the cells trypsinized, and appropriate numbers of cells were seeded in 60-mm dishes. After incubation for 10 days, clonogenic survival was determined by counting crystal violet–stained colonies consisting of more than 50 cells. The surviving fractions were calculated from three independent experiments.

### Flow-cytometric analysis

After treatment with KPU-300 and/or X-irradiation, collected culture medium and trypsinized cells were centrifuged together. After washing, the pellets were fixed in 4% paraformaldehyde for 30 min. Cells were subsequently stained with a monoclonal antibody against phospho-histone H3 (Ser10) (1:50) (Cell Signaling Technologies) for 1 h at room temperature, and then with Alexa Fluor 647–conjugated anti-rabbit IgG (1:500) for 30 min. Finally, cells were washed in phosphate-buffered saline (PBS), and re-suspended in PBS containing 10 μg/mL Hoechst 33342 dye solution (Invitrogen, Carlsbad, CA) to examine DNA content, and incubated for at least 30 min. Cells were then re-washed and stored in ice-cold PBS. Before analysis, single-cell suspensions were strained through nylon mesh. Samples were analyzed using a FACSCanto II (BD Bioscience, Franklin Lakes, NJ) with the FlowJo software (Tree Star, Ashland, OR).

### Spheroid formation

A Hydrocell^TM^ 96-well plate (CellSeed, Tokyo, Japan) was used to generate spheroids. Three hundreds HeLa-Fucci cells were plated onto each well and incubated for 7 days. After the spheroids became visible to the naked eye during incubation, the culture medium was exchanged with fresh medium every 2 days. Spheroids around 500 μm in diameter were transferred to the agar-spread culture dish containing growth medium, which was originally prepared for observation by the FV10i-LIV confocal laser scanning microscope (Olympus, Tokyo, Japan).

### Cell sorting

To collect cells in early M phase, mitotic shake-off method and cell sorting were combined as previously reported [35]. Briefly, to detach the loosely adhering mitotic cells, cells grown in flasks were shaken and rinsed gently about 10 times in DMEM. To purify only cells in early M phase, the green cell fraction with a high intensity of green fluorescence was isolated using a cell sorter MoFlo XDP (Beckman Coulter, Brea, CA). All of these processes were performed at ice-cold temperatures.

### Statistical analysis

Mean values were statistically compared using one way ANOVA with post hoc Tukey’s multiple comparison test or the two-tailed t-test. *P* values < 0.05 were considered statistically significant. The data are represented as means and standard error of the mean from three independent experiments.

## Results

### Characterization of cell cycle kinetics and cell survival in HeLa-Fucci cells following KPU-300 treatment

**Fig 1:**
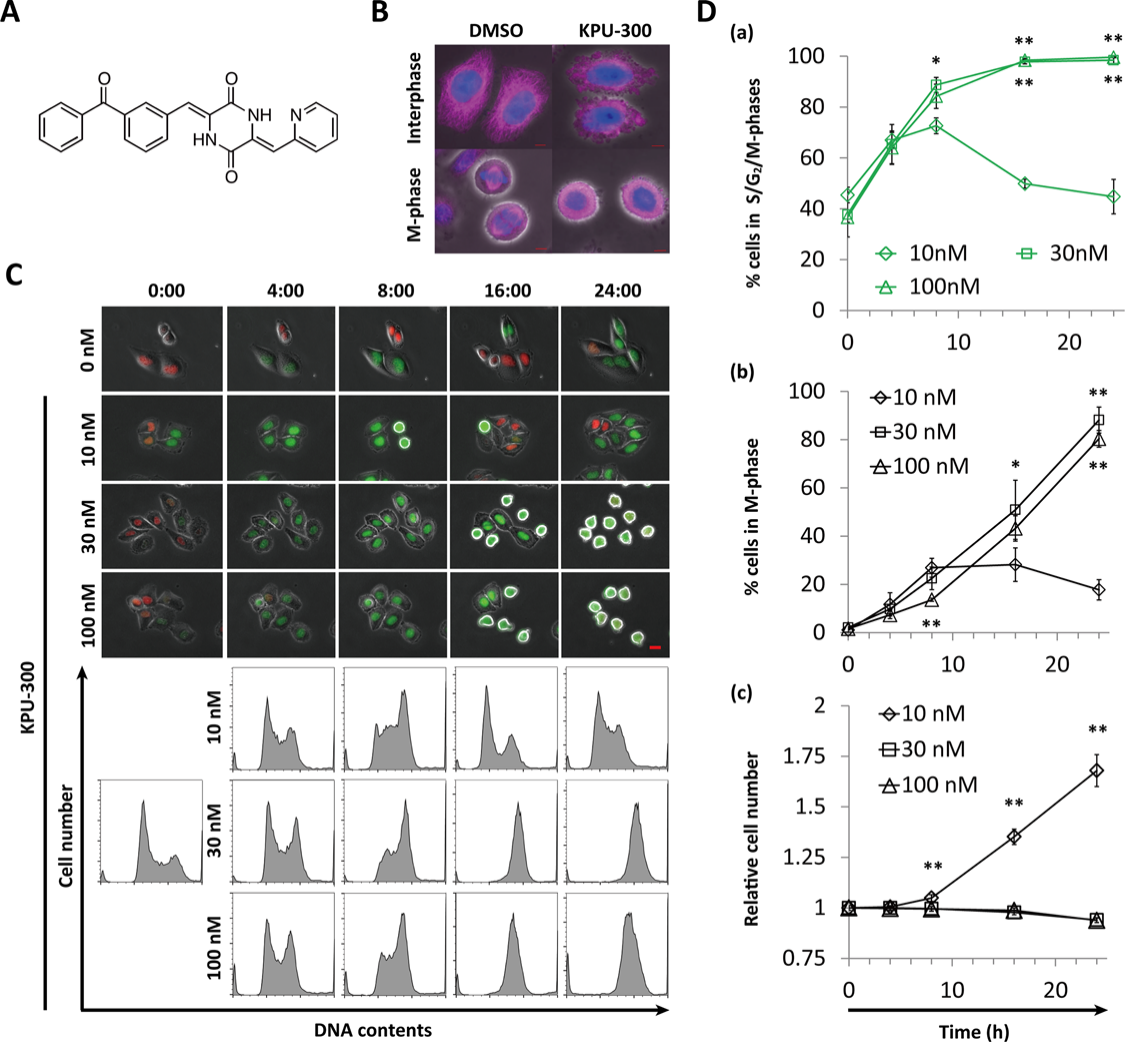
Characterization of cell cycle kinetics in HeLa-Fucci cells following KPU-300 treatment. (A) Chemical structure of KPU-300. (B) Immunostaining for β-tubulin. Exponentially growing HeLa-Fucci cells were fixed and prepared for immunostaining following treatment with 30 nM KPU-300 for 16 h. Blue, DAPI; pink, β-tubulin. Bar, 5 μm. (C) Time course of Fucci fluorescence and histogram of DNA content following KPU-300 treatment. Cells were treated with the indicated concentrations of KPU-300 and prepared for time-lapse imaging and flow-cytometric analysis. The time points are shown as hours:minutes in each image; 0:00 represents the start of drug treatment. Bar, 20 μm. (D) Time course of the percentages of green fluorescent cells (a), M-phase cells (b), and total cell number (c) following KPU-300 treatment. Green cells and M-phase cells were manually counted in merged fluorescence and phase contrast images. A total of 170–350 cells obtained from 8–11 visual fields were counted in one experiment. M phase cells adopt a round-shape, accompanied by disappearance of the nuclear envelope. Data represent means ± S.E. of values from three independent experiments. *, *p* < 0.05; **, *p* < 0.01 vs. treatment with 10 nM for the same duration of time.

Fig 1A shows a schematic representation of the chemical structure of KPU-300, a novel anti-microtubule agent that possesses a unique 2-pyridyl structure [16]. When cells were treated with 30 nM KPU-300, microtubule structures were disrupted and the mitotic spindle was not formed (Fig 1B), both characteristic consequences of treatment with inhibitors of microtubule polymerization [36–38]. We next examined the kinetics of Fucci fluorescence in HeLa-Fucci cells following treatment with various concentrations of KPU-300 (Fig 1C, upper panel). In the Fucci system, cells in G1 and S/G2/M phases basically emit red and green fluorescence, respectively, whereas cells in early S phase emit both [34]. Images of the cells shown in Fig 1C, acquired after longer incubation at 30 nM, are also shown in S1 Fig. Because cells collapsed after 24 h of drug treatment, in subsequent experiments we restricted the observations to time points prior to 24 h. The kinetics corresponded to those of G2/M fractions detected by flow-cytometric analysis (Fig 1C, lower panel). At a drug concentration of 10 nM, the percentage of green cells gradually increased, reaching a maximal value of ~70%, and subsequently decreased to the control level (~40%). On the other hand, at higher concentrations (30 and 100 nM), the percentage continued to increase up to 24 h after treatment, reaching almost 100% (Fig 1Da)(S1 Table). Close kinetics were also obtained in the percentage of M-phase cells, indicating that cells were almost completely arrested in M phase following KPU-300 treatment at concentrations ≥ 30 nM (Fig 1Db) (S1 Table). Accordingly, increase in cell number was completely inhibited at concentrations > 10 nM (Fig 1Dc) (S1 Table). Interphase and M phase cells could be distinguished easily: M phase cells have a characteristic round shape, accompanied by disappearance of the nuclear envelope; fluorescence in interphase cells is localized to the nucleus, but is spread throughout the whole cell in M phase cells (c.f. images of cells dispersed from the spheroid, as shown below) [34].

**Fig 2:**
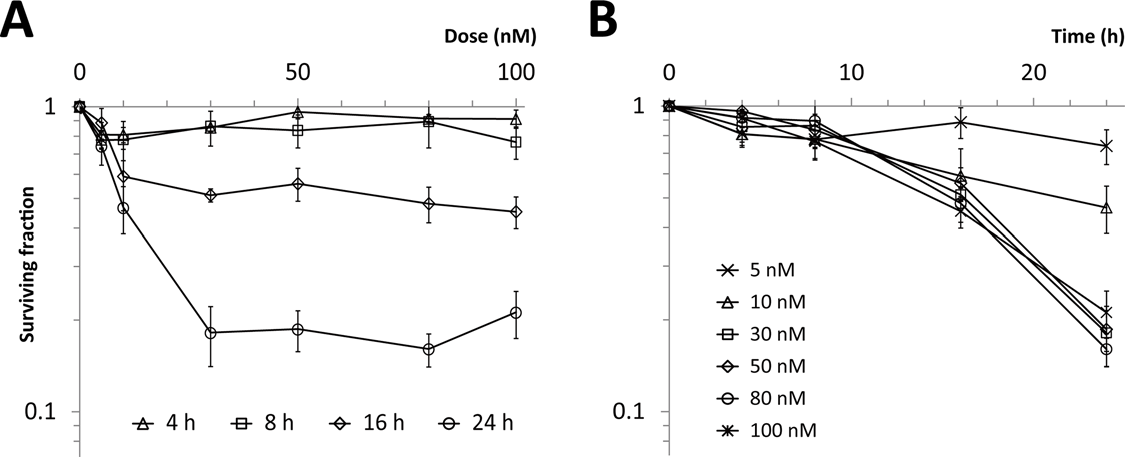
Cell survival curves following KPU-300 treatment. (A) Dose dependency of surviving fractions after KPU-300 treatment for the indicated times. (B) Time course of surviving fractions after KPU-300 treatment at the indicated concentrations. After treatment with KPU-300, cells were prepared for colony-forming assays. Data represent means ± S.E. of values obtained from three independent experiments.

Next, we examined cell survival in colony-forming assays. Up to 8 h after treatment, no significant decrease in surviving fraction was detected at concentrations from 5 to 100 nM, whereas more than 16 h after treatment, a significant decrease was observed at concentrations > 5 nM. Dose dependence was not detected in the range from 30 to 100 nM (Fig 2)(S2 Table). Similar results were obtained in other human tumor cell lines using the CCK-8 assay (S2 Fig)(S3 Table), confirming that the observed properties were not unique to HeLa-Fucci cells.

### Abnormal fluorescence in M phase is detectable in HeLa-Fucci cells following KPU-300 treatment

**Fig 3:**
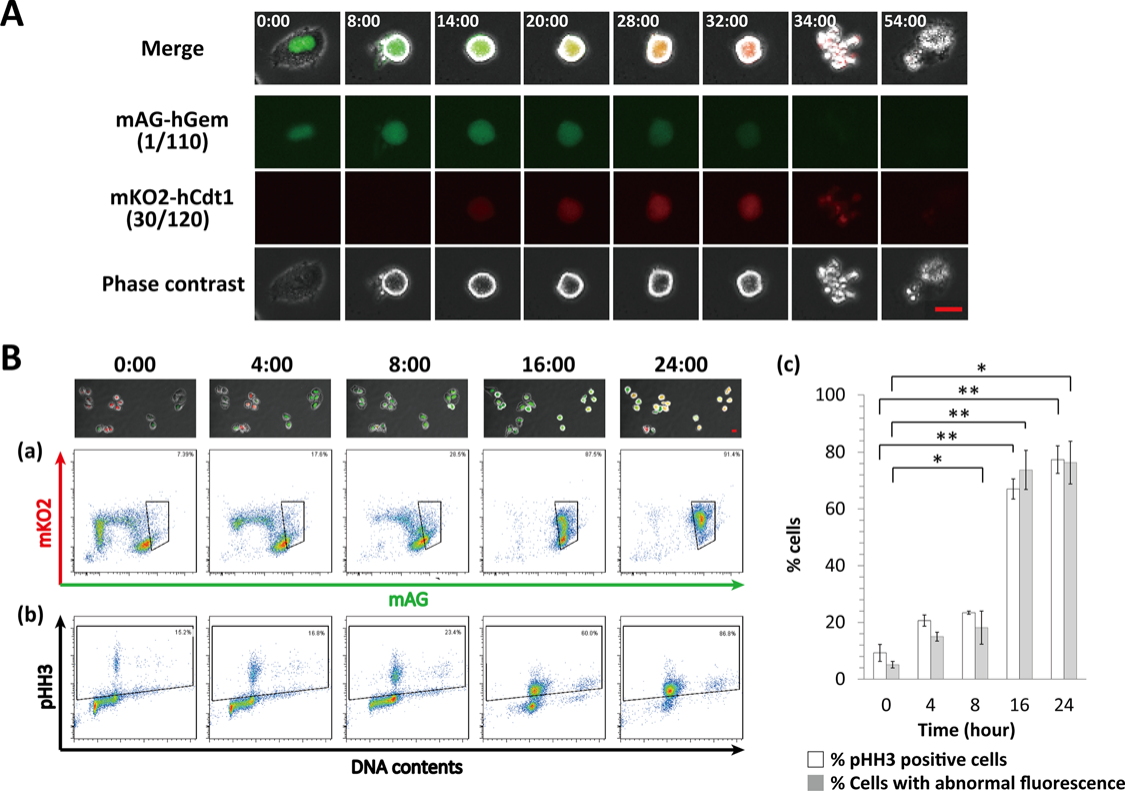
Characterization of abnormal Fucci fluorescence following KPU-300 treatment. (A) Representative images of abnormal fluorescence after treatment with KPU-300. The time points are shown as hours:minutes in each image; 0:00 represents the start of drug treatment. Bar, 20 μm (B) Relationship between abnormal Fucci fluorescence and M phase following KPU-300 treatment. (a) Two-dimensional flow-cytometric analysis of Fucci fluorescence. The area within a quadrangle represents cells expressing abnormal Fucci fluorescence. (b) Two-dimensional flow-cytometric analysis of DNA content and phosphorylated histone H3 (pHH3). The area within a quadrangle represents cells in M phase. The acquired time points are shown as hours:minutes in each image; 0:00 represents the start of drug treatment. (c) Quantitative analysis of cells with abnormal Fucci expression and those in M phase in Fig 3a and 3b. Data represent means ± S.E. of values obtained from three independent experiments. *p < 0.05; **p < 0.01 vs. controls at time 0.

We previously reported that plinabulin, which has colchicine-like microtubule depolymerization activity, induces abnormal fluorescence in M-phase HeLa-Fucci cells, and that this is an indicator of mitotic catastrophe [39]. In this study, we obtained similar results following treatment with 30 nM KPU-300. During the initial stage of mitosis, only green fluorescence was observed, but abnormal red fluorescence gradually became detectable; subsequently, the cells underwent mitotic catastrophe (Fig 3A). In flow-cytometric analysis, the green fraction gradually shifted upward, becoming double-positive for green and red fluorescence, including most cells after 24 h of treatment (Fig 3Ba). The double-positive fraction was confirmed to be in M phase by immunostaining for phosphorylated histone H3 (Fig 3Bb). A very small fraction of cells showing polyploidy are originally included in HeLa-Fucci cells, which were detected in Fig 3Bb. It is likely that such cells also apparently showed the phosphorylated form of histone H3 after 16 h of treatment time. Quantitative analysis also supported these results (Fig 3Bc)(S4 Table). Considering that the percentage of sub–G1 phase cells (apoptotic fraction) was 16.8% (data not shown) after 24 h KPU-300 treatment, we conclude that almost all cells were arrested in M phase with abnormal fluorescence.

### Following KPU-300 treatment, cells in spheroids exhibit cell cycle kinetics similar to those in monolayer cultures

**Fig 4:**
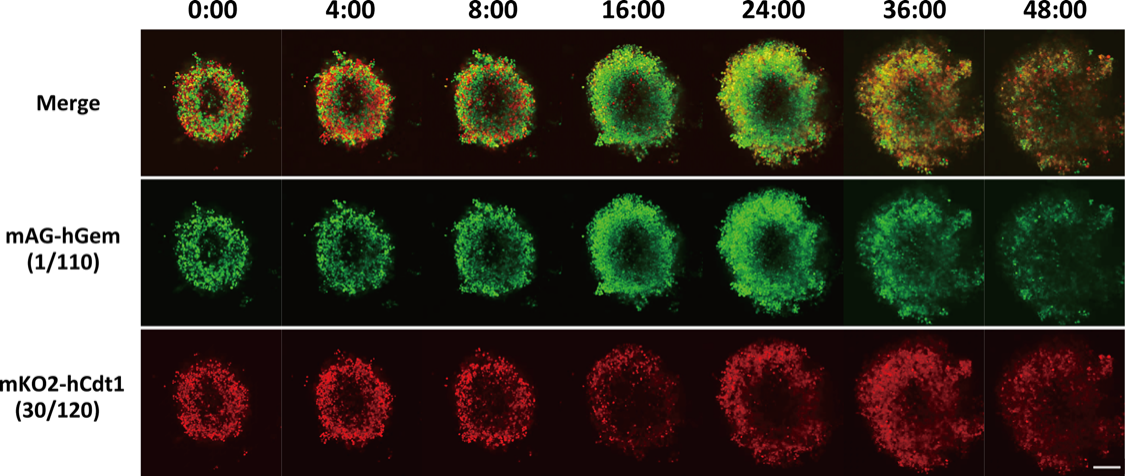
Confocal fluorescence imaging of spheroids after treatment with KPU-300. The spheroid was treated with 30 nM KPU-300 and observed at the indicated times at the depth of 65 μm from the bottom using the confocal laser scanning fluorescence microscopy. The time points are shown as hours:minutes in each image. Bar, 200 μm.

We next examined cell cycle kinetics of cells in spheroids following KPU-300 treatment. We previously reported that HeLa-Fucci cell spheroids with a diameter of ~500 μm have an outer growth fraction ~80 μm thick and an inner quiescent fraction. However, it was impossible to visualize the latter fraction under live conditions due to the optical limitations of the confocal fluorescence microscope [40]. Outer cells visualized at a depth of 65 μm from the spheroid surface responded to the KPU-300 treatment, as shown in Fig 4: the number of green cells increased gradually, and that of red cells increased 24 h after the treatment. Both signals reached a peak around 28 h after the treatment (data not shown), and fluorescence intensity gradually decreased thereafter. After drug treatment, the size of the spheroids significantly increased, indicating that cell-cell contact was loosened. When the spheroids were dispersed 24 h after the treatment, most of the cells were yellow (i.e., M phase) cells lacking a nuclear envelope, in contrast to the interphase cells from the untreated spheroid, in which fluorescence was localized in the nucleus (S3 Fig). These results suggest that the drug treatment loosened cell-cell contact, leading to recruitment of the quiescent fraction to growth fraction, which subsequently responded to the drug. As a control, fluorescence images of the untreated spheroid 24 h obtained under exactly the same observation conditions used for the KPU-300-treated spheroid are shown in S4 Fig.

### Combined treatment of X-irradiation following KPU-300 treatment significantly radiosensitizes HeLa-Fucci cells

**Fig 5:**
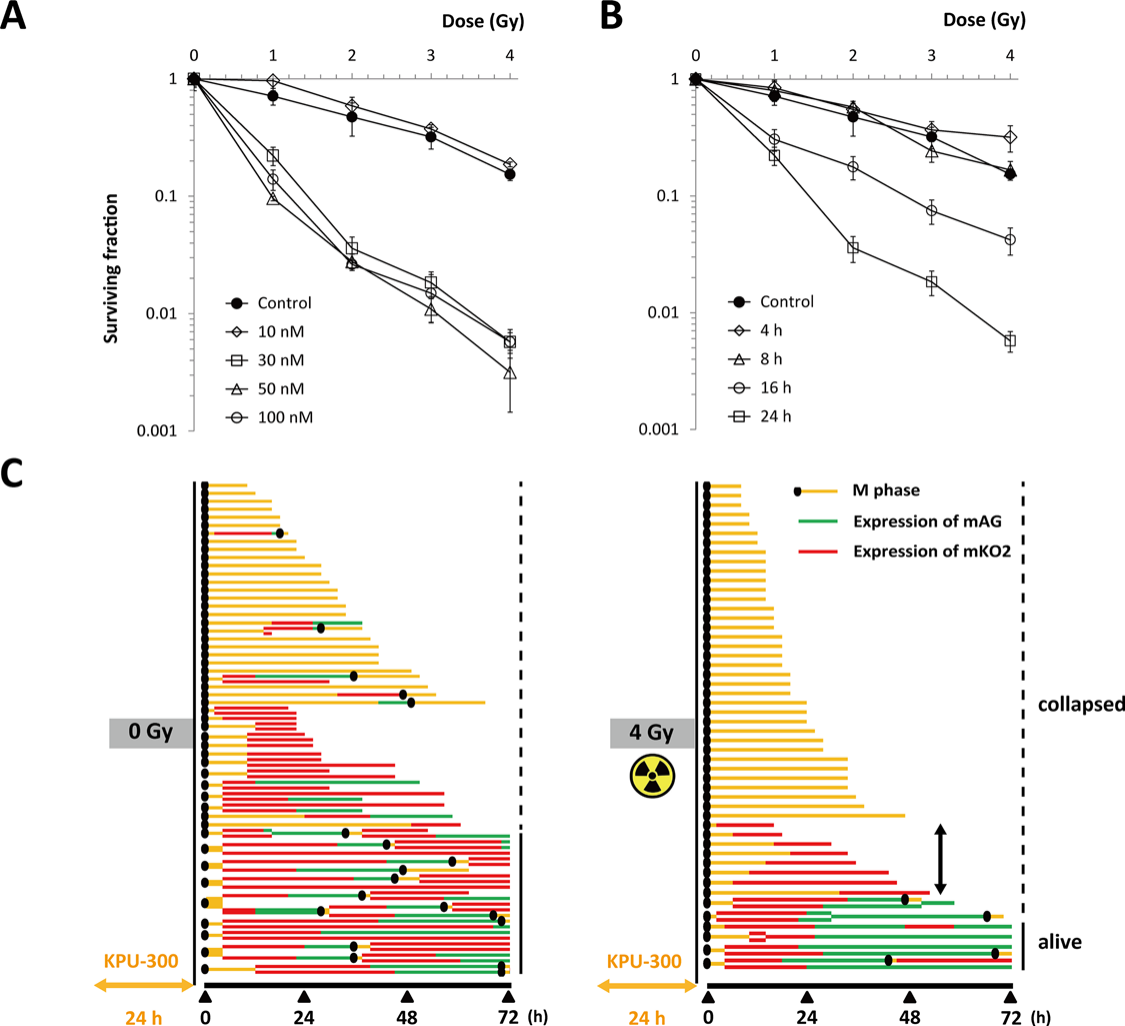
Radiosensitization of HeLa-Fucci cells by the sequence of irradiation after treatment with KPU-300 and their pedigree analysis. (A)Survival curves of HeLa-Fucci cells irradiated after treatment with variousconcentrations of KPU-300 for 24 h (left panel). Survival curves in HeLa-Fucci cells irradiated after treatment with 30 nM KPU-300 for various times (right panel). Surviving fractions (SFs) were determined by colony-forming assay. SFs were normalized such that SFs in the absence of irradiation had a value of 1. Data represent means ± S.E. of values obtained from three independent experiments. (B) Pedigree analysis in cells treated with KPU-300 alone (left panel) or in combination with irradiation (right panel). After treatment with 30 nM KPU-300 for 24 h, the medium was replaced by fresh medium without drug. Time-lapse imaging was performed in the presence or absence of 4 Gy irradiation, starting immediately after drug treatment and continuing for up to 72 h. Lines extending to the right end of the panel represent cells surviving for at least 72 h, and shorter lines represents cells that collapsed within 72 h; the position of the end of the line indicates the time of cell collapse. A unique cell death mode showing anaphase skipping was observed albeit with an infrequent event in irradiated cells following drug treatment (bidirectional arrow).

Because KPU-300 synchronized cells in M phase, the most radiosensitive stage of the cell cycle, we reasoned that cells would be radiosensitized following KPU-300 treatment. After 24 h treatment with various concentrations of KPU-300, cells were X-irradiated, and survival curves were obtained (Fig 5A)(S5 Table). As shown in Fig 2, the surviving fractions after 24 h treatment with KPU-300 alone at concentrations ≥ 30 nM were ~20%. Synergistic radiosensitization was observed for cells after treatment at ≥ 30 nM, whereas at 10 nM, only an additive effect was observed. When the concentration was fixed at 30 nM, synergistic effects were observed only when drug treatment duration was ≥ 16 h (Fig 5B)(S5 Table).

We next investigated how cells died after drug treatment (30 nM, 24 h) with or without X-irradiation (4 Gy). Pedigree analysis was performed after removal of the drug (Fig 5C). After drug treatment alone, ~50% of the cells underwent mitotic catastrophe following abnormal fluorescence (Fig 3), and ~25% of cells survived for up to 72 h after the start of observation (Fig 5C, left panel). In the typical pattern of mitotic catastrophe, cells exhibited both green and red fluorescence until collapse. After a combination of 24 h drug treatment and irradiation, ~80% of cells underwent mitotic catastrophe, and ~10% survived for up to 72 h after the start of observation (Fig 5C, right panel). Following combined treatment, a small fraction of cells died in G1 phase after skipping anaphase; however, this infrequent event was unlikely to explain the remarkable radiosensitization we observed. Time-lapse imaging and pedigree analysis for M phase cells receiving only 4 Gy are shown in S4 Fig as a control.

### Synchronization of cells at early M phase is a mechanism of synergistic radiosensitization after drug treatment

**Fig 6:**
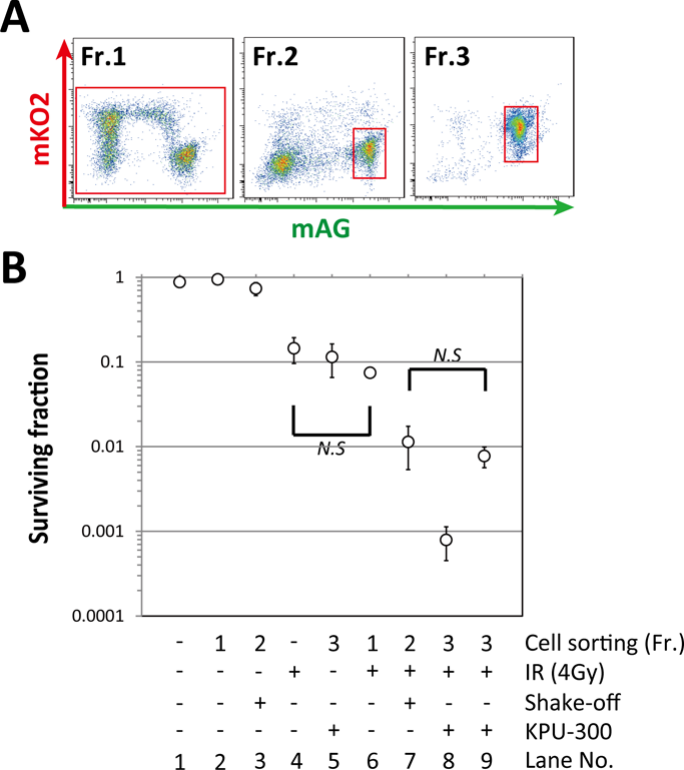
Radiosensitivity in cells in early M phase is comparable to that in KPU-300–treated cells. (A) Fractions sorted by flow cytometry. Fr. 1, whole cell population; Fr. 2, cell fraction enriched in early M phase following the shake-off method; Fr. 3, cell fraction accumulated in early M phase following KPU-300 treatment for 24 h. (B) Radiosensitivity in each cell fraction following various treatments. Radiation dose was 4 Gy, and concentration of KPU-300 was 30 nM. A value of “-” in the “Fr.” row (i.e., lanes 1 and 4) indicates that cell sorting was not performed. The SF for Lane No. 9 was normalized by dividing the SF for Lane No. 8 by that for Lane No. 5. Data represent means ± S.E. of values obtained from three independent experiments. Error bars are not displayed when they would have been smaller than the circular symbol indicating the mean. *N*.*S*., not significant by either ANOVA or t-test.

Judging from the relationship between cell cycle kinetics after drug treatment and radiosensitization patterns of survival curves, we reasoned that the underlying radiosensitizing mechanism of KPU-300 involved synchronization of cells at M phase. To test this idea, we tried to collect M-phase cells by shake-off method [25]. However, when the collected cells were examined by flow-cytometric analysis, the green cells were contaminated with many cells that emitted no fluorescence (representing early G1 cells), as previously reported [35]. Therefore, we were unable to use the cells collected using the shake-off method alone. Taking advantage of chromosome visualization by GFP-histone H2B, we found previously that plinabulin stops the cell cycle around metaphase [39]. Fr. 2 in Fig 6A, acquired from cells obtained by the shake-off method plus cell sorting, contained more than 80% cells in early M phase (i.e., prometaphase and metaphase) in our previous report [35]. Therefore, we predicted that the surviving fraction of cells in Fr. 2 would provide a key solution if the radiosensitization is truly due to synchronization of cells at the specific phase. Hence, we determined surviving fractions (SFs) after various treatments, as shown in Fig 6B. The shake-off and cell sorting procedures did not significantly affect radiosensitivity. The SF of cells in the whole population following KPU-300 treatment for 24 h or irradiation with 4 Gy was ~0.1 (Lane No. 5 and 6). By contrast, the SF of early M-phase cells in Fr. 2 following 4 Gy irradiation was much lower, ~0.01 (Lane No. 7). This observation was consistent with our previous results [35]. When cells were subjected to 4 Gy irradiation after KPU-300 treatment for 24 h, the SF was ~0.001 (Lane No. 8). Normalizing for the effect of KPU-300 treatment alone, i.e., the SF for Lane No. 8 was divided by that for Lane No. 5, the SF became ~0.01 (Lane No. 9), which coincides with the SF of early mitotic cells. Thus, the radiosensitizing effect of the KPU-300 treatment is consistent with radiosensitization obtained by synchronizing cells in early M phase, and the radiosensitizing effect of KPU-300 can be attributed to cell cycle synchronization.

### KPU-300 treatment after X-irradiation exerts an additive effect in low-dose irradiation, but an antagonistic effect in high-dose irradiation

**Fig 7:**
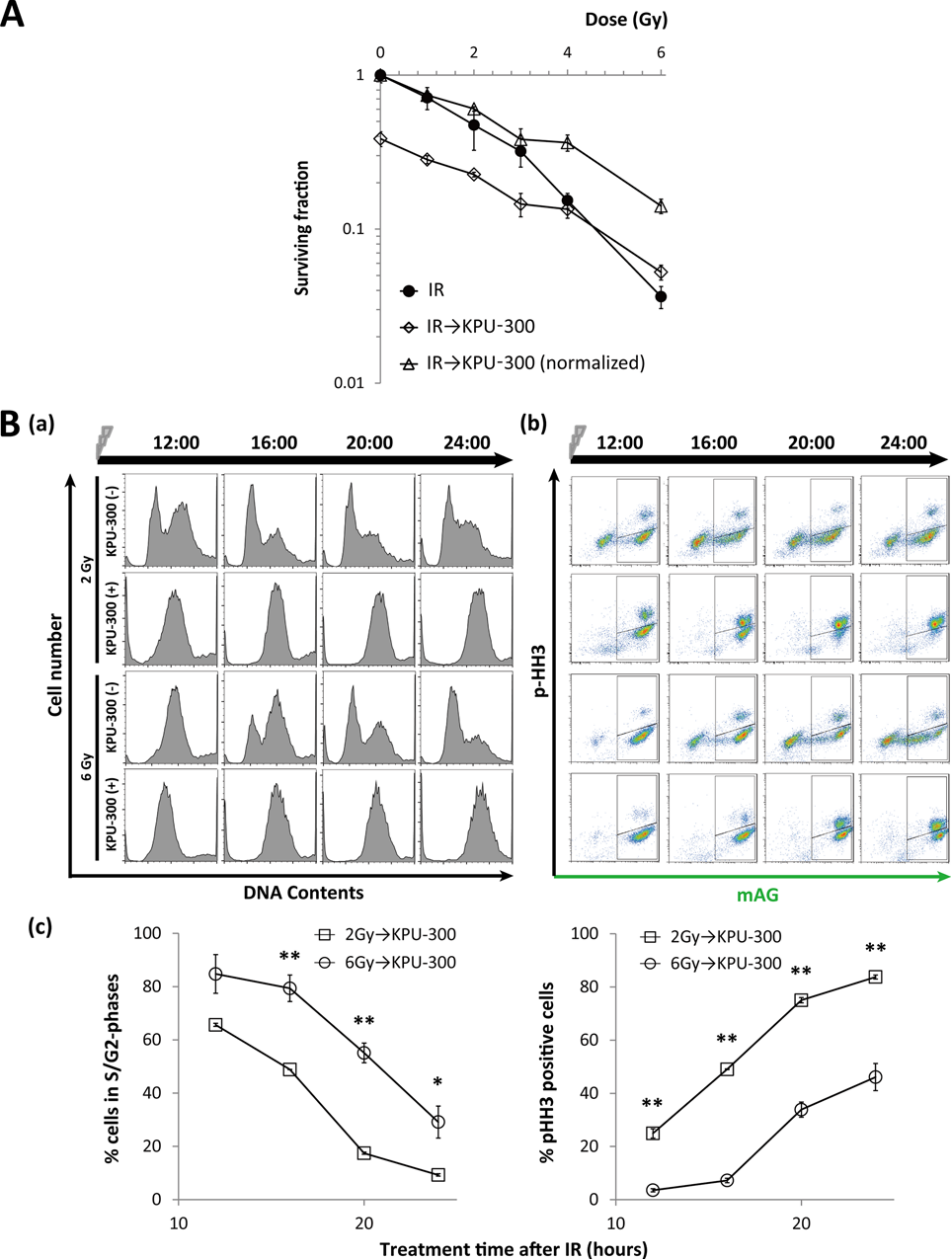
Radiosensitivity and cell cycle kinetics of cells subjected to KPU-300 treatment after irradiation. (A) Survival curves in HeLa-Fucci cells treated with KPU-300 after irradiation. Cells were treated with 30 nM KPU-300 for 24 h immediately after irradiation, and then prepared for colony-forming assay. For normalization, the curve for combined treatment was shifted upward so as to obtain the surviving fraction 1 at 0 Gy. Data represent means ± S.E. of values obtained from three independent experiments. (B) Cell cycle kinetics after the same treatment described in Fig 7A. (a) Time course of DNA content with or without KPU-300 treatment after 2 Gy or 6 Gy irradiation. (b) Time course of two-dimensional flow-cytometric analysis to detect green fluorescence and an M-phase marker. The acquired time points are shown as hours:minutes in each image. (c) Quantitative analysis of green cells (left panel) and M-phase cells (right panel) after the same treatment described in Fig 7A. Data represent means ± S.E. of values obtained from three independent experiments. *, *p* < 0.05; **, *p* < 0.01 vs. lower values for the same time points.

We next examined the effect of reversing the combination sequence, i.e., by treating cells with KPU-300 for 24 h immediately after X-irradiation (Fig 7A)(S6 Table). At doses < 4 Gy, the SFs exhibited an additive effect, whereas at doses ≥ 4 Gy, they exhibited an antagonistic effect. Thus, this sequence of combination treatment did not result in synergistic radiosensitization. This observation is reasonable given that the observed radiosensitization could be attributed to synchronization in early M phase, as described above. To investigate the mechanism of the antagonistic effect at high doses of irradiation, we closely inspected the cell cycle kinetics (Fig 7B). DNA content analysis by flow cytometry revealed that cells were released later from G2 arrest after 6 Gy irradiation than after 2 Gy irradiation; however, when irradiation was combined with KPU-300 treatment, no useful information was obtained to explain the difference (Fig 7Ba). The elongation of the green phase represents G2 arrest [41]; therefore, we concluded that G2 arrest was more strongly induced during KPU-300 treatment following 6 Gy irradiation than following 2 Gy irradiation (Fig 7Ba). Consequently, the ratio of mitotic cells released from G2 arrest was significantly higher in cells exposed to 2 Gy than in those exposed to 6 Gy (Fig 7Bb, Fig 7Bc)(S6 Table). Thus, elongated G2 arrest results in a smaller proportion of mitotic cells during the limited period of KPU-300 treatment (24 h), leading to decreased cytotoxicity by KPU-300.

## Discussion

Because colchicine-type anti-microtubule drugs are mostly vascular disrupting agents (VDA) [31–33, 42–44], we closely examined their effect on cell cycle kinetics and investigated the possibility of using such agents as a radiosensitizer *in vitro*. Specifically, using the novel colchicine-type anti-microtubule agent KPU-300, we characterized the detailed cell-cycle kinetics following KPU-300 treatment by taking advantage of the Fucci system. We observed a dramatic radiosensitization when irradiation was administered after KPU-300 treatment at concentrations ≥ 30 nM for ≥ 16 h. Under these conditions, most of the cells were in M phase at the time of irradiation.

Tishler et al. reported that paclitaxel induces dramatic radiosensitization 24 h after irradiation, but not 2 or 8 h after irradiation [45]. This timing is well correlated with the increase in the G2/M fraction, representing a relatively radiosensitive phase of the cell cycle, as determined by flow-cytometric analysis. Because cells in late S phase are the most radioresistant, and the duration of G2/M phase is relatively short, radiosensitivity changes dramatically during a short cell cycle progression. Therefore, to precisely determine the effect of anti-microtubule agent–induced synchronization on radiosensitization, a much more elaborate study was essential. In our previous study, we visualized chromatin configuration in cells expressing GFP-histone H2B after plinabulin treatment, and determined that the cells were synchronized around metaphase [39]. Thus, we had to specifically validate whether KPU-300–induced radiosensitization could be explained by the radiosensitivity of cells in early M phase (i.e., around metaphase). This consideration prompted us to collect mitotic cells by the shake-off method, originally reported by Terasima and Tolmach; this method takes advantage of the loose attachment of mitotic cells to the substrate and enables specific enrichment of mitotic cells by simple shaking and washing of culture dishes [25, 26]. In their hands, the purity of mitotic cells was 85–90% for the HeLa-S3 cell line [27]. However, Sinclair and Morton reported that such a simple shake-off method does not work for Chinese hamster cells [46]. Likewise, we found that the method did not work for HeLa-Fucci cells, and that the samples obtained were massively contaminated with cells in early G1 phase (Fig 6A). In our previous work, it was possible to isolate cells in early G1 phase, late M phase, and early M phase by combining the shake-off method with sorting based on the properties of the Fucci system; the resultant fraction of early M-phase cells contained more than 80% of cells in prometaphase and metaphase [35]. Thus, the Fucci system allowed us to determine whether the extent of radiosensitization induced by KPU-300 could be explained by the radiosensitivity of isolated early M-phase cells. Indeed, this was the case (Fig 6). The molecular mechanisms underlying the highest radiosensitivity in mitotic cells remain largely unclear; however, previous work has shown that chromatin in mitotic cells is more susceptible to DNA double-strand breaks (DSBs) than that in interphase cells [47], and mitotic cells are deficient in the late stage of the DSB repair process [48].

The surviving fraction (SF) of cells following treatment with 30 nM KPU-300 for 24 h was in the range 0.1–0.2. Our results indicate that cells that survived the treatment exhibited dramatic radiosensitization in early M phase. After drug treatment alone, the typical mode of cell death was mitotic catastrophe. Even after a combination of drug treatment with irradiation, it was likely that the incidence of the cell death mode was enhanced, although a subtle incidence of anaphase slippage was observed for at least 72 h after irradiation. In addition to M-phase synchronization, inhibition of DNA repair has also been proposed as a radiosensitizing mechanism [49]. Poruchynsky et al. reported that disruption of intracellular trafficking of DNA repair proteins from the cytoplasm into the nucleus is an important mechanism underlying radiosensitization [50]. Given that the nuclear envelope is not present in M phase, however, this mechanism is not applicable to mitotic cells. This concept supports our results that combined treatment did not induce more radiosensitization than expected of cells in early M phase. Further molecular studies should be performed to elucidate these issues. Nonetheless, our methodology using the Fucci system verified for the first time the mechanism of radiosensitization from the viewpoint of cellular radiosensitivity. Notably, an antagonistic effect was obtained when cells were treated with KPU-300 immediately after irradiation at high doses. During a limited period of drug treatment, the duration of G2 arrest is likely to affect the cytotoxicity of KPU-300, i.e., the duration of mitotic delay is crucial for the cytotoxicity of anti-microtubule agents.

Using the Fucci system, Yano et al. elegantly demonstrated that L-methioninase [51] or Salmonella typhimurium A1-R [52], which is able to induce G0/G1 tumor cells to advance into S/G2 phase, can sensitize tumor cells to DNA-interacting or DNA synthesis-inhibiting chemotherapeutic drugs such as cisplatin, doxorubicin, and 5-fluorouracil. The resultant changes in cell cycle phase are clearly indicated by a fluorescence shift from red to green. Considering that M is the most radiosensitive phase of the cell cycle and late S is the most radioresistant, progression from G0/G1 to S/G2 is unlikely to be sufficient for sensitization; rather, synchronizing in early M phase (e.g., by KPU-300 treatment) would be an ideal radiosensitizing strategy.

As noted above, colchicine-type anti-microtubule agents also have vascular disrupting functions, resulting in rapid necrosis in tumor tissue via blocking tumor blood flow [12, 32, 33, 42, 43], leading to hypoxia in tumor tissue, which may cause radioresistance. Taken together with our results, these findings may suggest that it will be quite difficult to determine the optimal timing of irradiation in combination with this class of drugs in a clinical setting. However, vascular disrupting agents (VDA) cause necrosis only in inner regions of solid tumors; recurrence arises from cells in peripheral regions, due to differences in the properties of tumor vessels in the inner and peripheral regions [53]. Given that tumor vessels in the peripheral regions are refractory to VDA, we speculate that the radiosensitizing effects of KPU-300 obtained in this study could be applied to peripheral regions. It is not necessary to consider VDA-induced hypoxia, because eradication of tumor cells in this inner region could be achieved by subsequent necrosis. Hypoxic tumor cells should exist even in peripheral regions, and such cells may be reoxygenated by elimination of oxygen-consuming tumor cells and/or loosening of cell–cell contacts, as demonstrated by our findings in spheroids (Fig 4), providing an opportunity for strong radiosensitization (i.e., reoxygenation, recruitment to the growth fraction, and synchronization in early M phase) to the next round of irradiation. Although paclitaxel has been reported to achieve such reoxygenation in whole tumor tissues [54, 55], here we propose, for the first time, the aforementioned radiosenstizing strategy using VDA. Given the information about oxygen tension and cell cycle kinetics via surrogate markers during the treatment, further optimal timings could be designed for the maximal tumor cell killing efficiency in an individualized way.

## Supporting Information

S1 Fig Fucci fluorescence images later times after 24 h of 30 nM KPU-300 treatment in Fig 1C. The time points are shown as hours:minutes in each image. Bar, 20 μm.
(TIF)

S2 Fig Cell viability assays performed on various tumor cell lines after treatment with the indicated concentrations of KPU-300. Cell viability was determined 24 h after treatment, as described in Materials and Methods. Data were normalized such that viabilities in the absence of treatment had a value of 1.
(TIF)

S3 Fig Fluorescence images dispersed from KPU-300-untreated and–treated spheroids. KPU-300-untreated (left panel) and -treated spheroids (30 nM, 24 h)(right panel) were gently physically dispersed and observed by a fluorescence microscope. Bar, 50 μm.
(TIF)

S4 Fig Confocal fluorescence imaging of untreated spheroids Spheroids were observed at varying depths from 36.9 μm to 88.5 μm, using confocal laser scanning fluorescence microscopy, 24 h under exactly the same observation conditions used for KPU-300-treated spheroids. Bar, 200 μm.
(TIF)

S5 Fig Fluorescence images in HeLa-Fucci cells irradiated at M phase. Time-lapse imaging for three cells irradiated (4 Gy) at M phase (upper panel). The time points are shown as hours:minutes in each image. Bar, 20 μm. Pedigree analysis for the three cells in the upper panel (lower panel). The colors and lines represent the same as those in Fig 5.
(TIF)

S1 Table Data points for Fig 1D. (XLSX)

S2 Table Data points for Fig 2. (XLSX)

S3 Table Data points for S2 Fig. (XLSX)

S4 Table Data points for Fig 3Bc. (XLSX)

S5 Table Data points for Fig 5A and 5B. (XLSX)

S6 Table Data points for Fig 7A and 7Bc. (XLSX)
