# Supplementary figures and images for: KPU-300, a Novel Benzophenone–Diketopiperazine–Type Anti-Microtubule Agent with a 2-Pyridyl Structure, Is a Potent Radiosensitizer That Synchronizes the Cell Cycle in Early M Phase
Source: PLoS One. 2015 Dec 30;10(12):e0145995. doi: 10.1371/journal.pone.0145995 (PMC4696839; doi:10.1371/journal.pone.0145995)

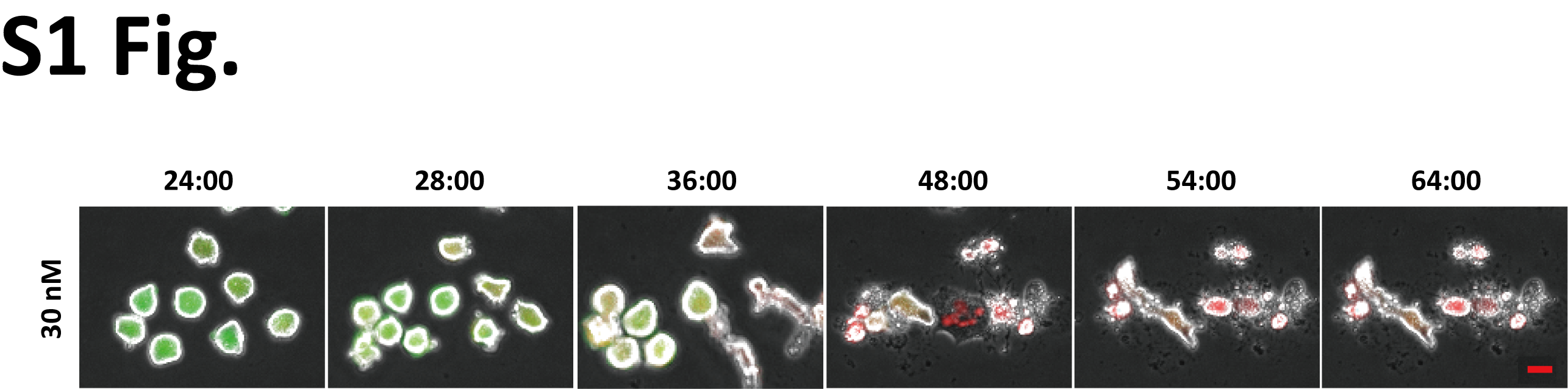

Supplement: S1 Fig — The time points are shown as hours:minutes in each image. Bar, 20 μm. (TIF) [file pone.0145995.s001.tif]

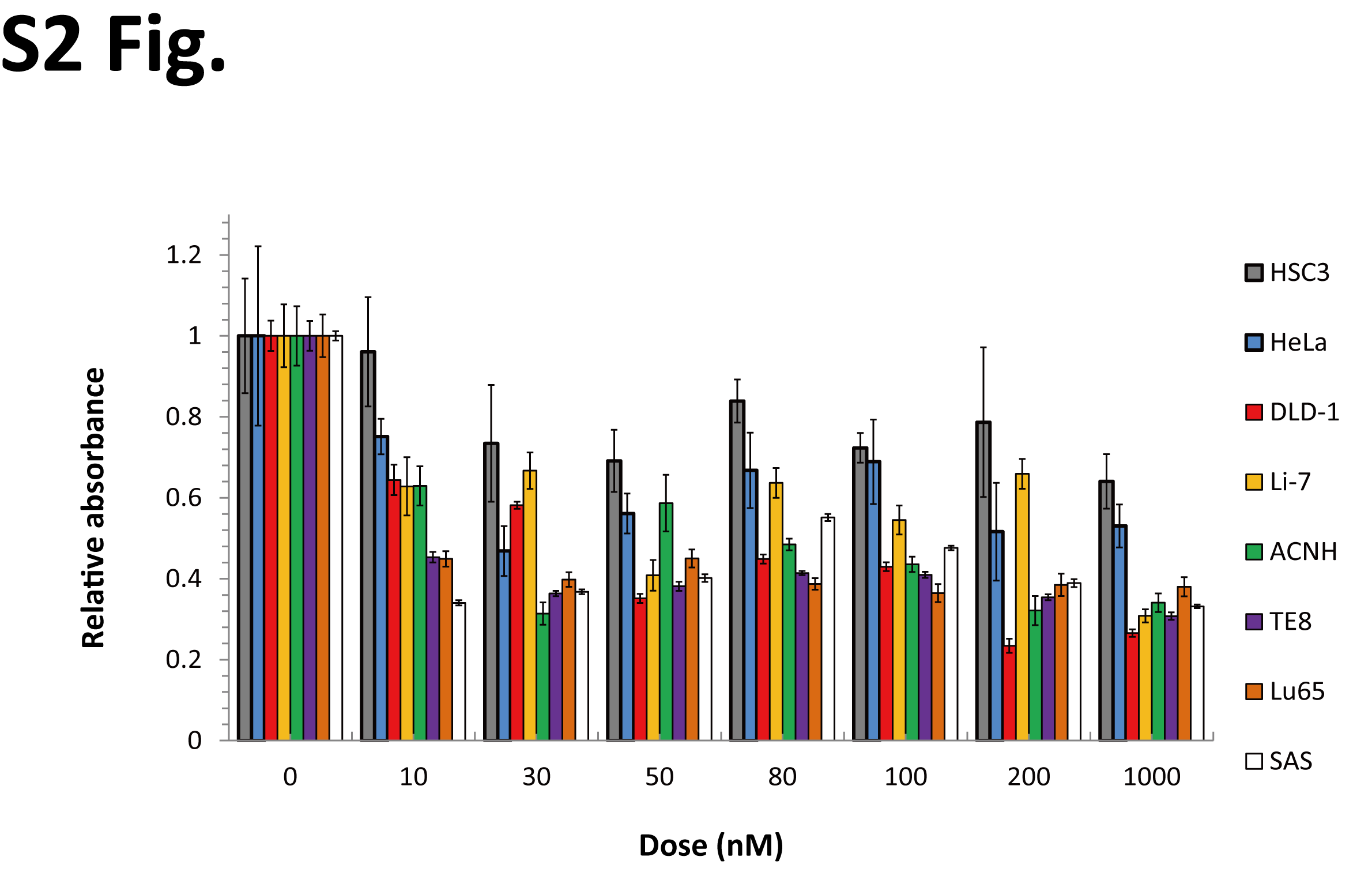

Supplement: S2 Fig — Cell viability was determined 24 h after treatment, as described in Materials and Methods. Data were normalized such that viabilities in the absence of treatment had a value of 1. (TIF) [file pone.0145995.s002.tif]

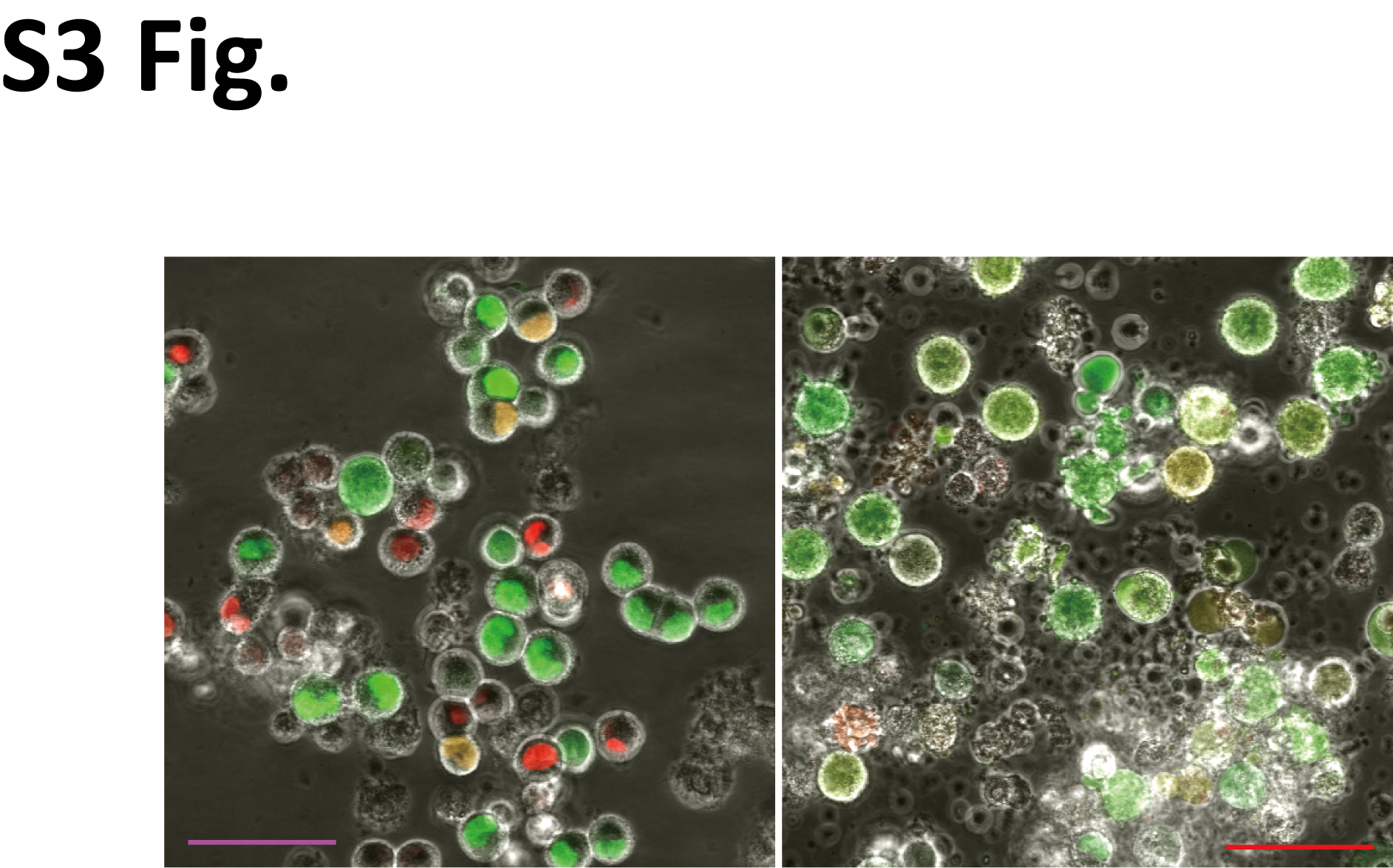

Supplement: S3 Fig — KPU-300-untreated (left panel) and -treated spheroids (30 nM, 24 h)(right panel) were gently physically dispersed and observed by a fluorescence microscope. Bar, 50 μm. (TIF) [file pone.0145995.s003.tif]

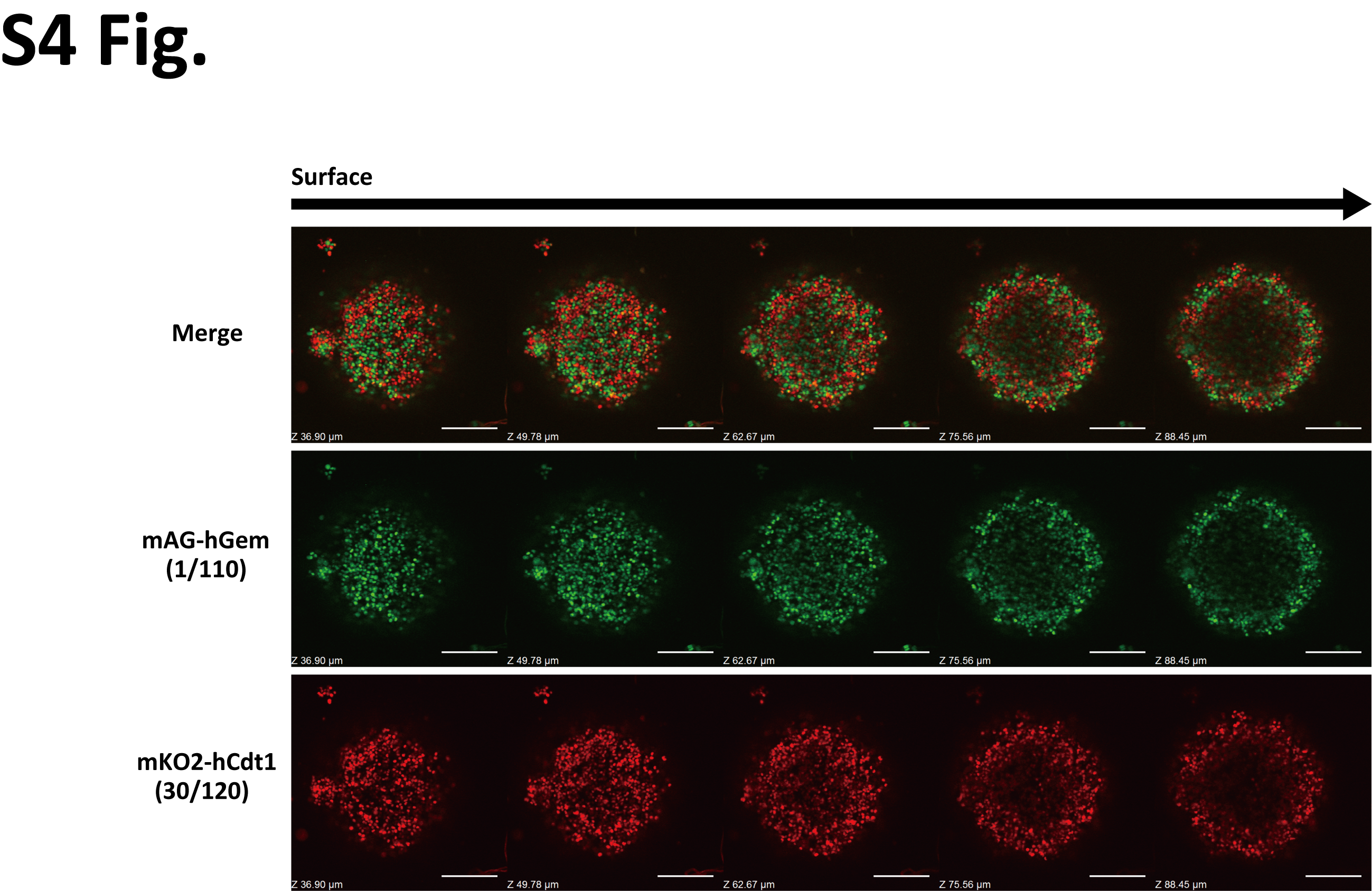

Supplement: S4 Fig — Bar, 200 μm. (TIF) [file pone.0145995.s004.tif]

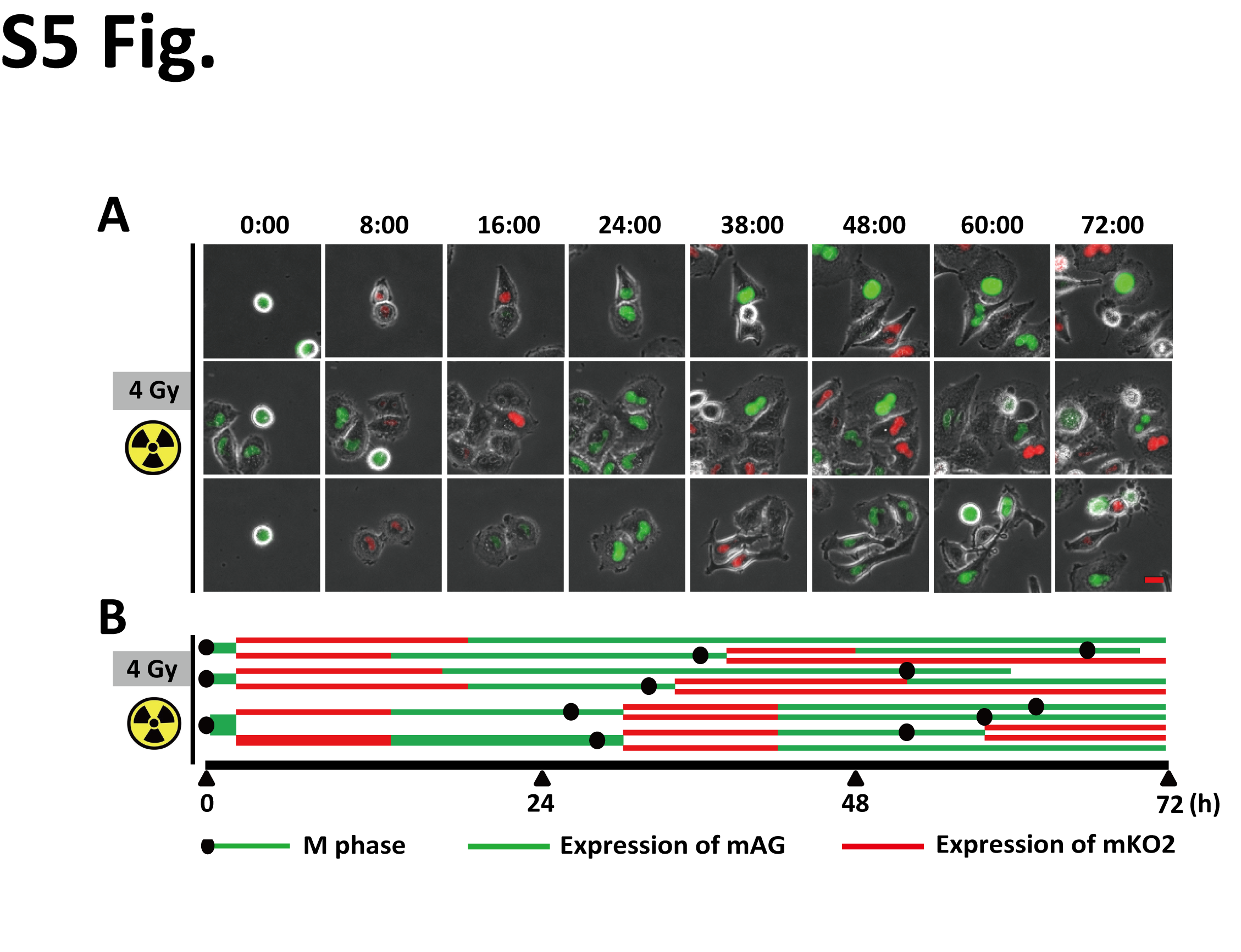

Supplement: S5 Fig — Time-lapse imaging for three cells irradiated (4 Gy) at M phase (upper panel). The time points are shown as hours:minutes in each image. Bar, 20 μm. Pedigree analysis for the three cells in the upper panel (lower panel). The colors and lines represent the same as those in Fig 5. (TIF) [file pone.0145995.s005.tif]
